# B_4_C–Graphene Nanoplatelet Composite Fabricated by Hot Pressing of Heterogeneously Co-Precipitated Powder Mixtures

**DOI:** 10.3390/ma19173592

**Published:** 2026-08-24

**Authors:** Aiyang Wang, Lanxin Hu, Li Zhu, Man Xu, Weimin Wang

**Affiliations:** 1 State Key Laboratory of Green and Efficient Development of Phosphorus Resources, Wuhan Institute of Technology, Wuhan 430205, China; aywang@whut.edu.cn; 2 Engineering Research Center of Environmental Materials and Membrane Technology of Hubei Province, School of Materials Science and Engineering, Wuhan Institute of Technology, Wuhan 430073, China; 3 School of Intelligent Transportation, Wuhan Business University, Wuhan 430056, China; 4 State Key Laboratory of Advanced Technology for Materials Synthesis and Processing, Wuhan University of Technology, 430070 Wuhan, China

**Keywords:** B_4_C, GNPs, heterogeneous co-precipitation, mechanical properties, mechanisms

## Abstract

Boron carbide (B_4_C) ceramics suffer from poor sinterability and inherent brittleness, which severely limit their engineering applications. In this work, B_4_C–graphene nanoplatelet (GNP) composites were fabricated by hot pressing using heterogeneously co-precipitated powder mixtures, with cetyltrimethyl ammonium bromide (CTAB) as a surfactant for achieving uniform dispersion of GNPs within the B_4_C matrix. The formation mechanisms of B_4_C–GNP hybrids were systematically elucidated. The results show that CTAB endows GNPs with positive charges, enabling electrostatic co-precipitation with negatively charged B_4_C particles to construct layered hybrid architectures. The GNP content has a significant modulation effect on the microstructure and mechanical properties of B_4_C composites. A maximum relative density of 99.65%, Vickers hardness of 33.5 GPa, and flexural strength of 488 MPa were obtained at 1 wt% GNPs, while the fracture toughness reached a peak value of 4.89 MPa·m^1/2^ at 2 wt% GNPs, representing a 63.5% improvement over monolithic B_4_C. The enhanced fracture toughness is attributed to multiple toughening mechanisms, including crack deflection, crack bridging, GNP pull-out, step-like fracture, and zigzag crack propagation. This study provides a feasible strategy for preparing uniformly dispersed ceramic–graphene composites with balanced mechanical properties.

## 1. Introduction

Boron carbide (B_4_C) is a high-performance structural ceramic material characterized by low density, high hardness, outstanding elastic modulus, superior wear resistance and high neutron absorption capability [1,2,3,4]. These excellent properties make B_4_C widely used in lightweight armor, cutting tools, wear-resistant components and nuclear shielding applications. However, the strong covalent bonds and low self-diffusion coefficient of B_4_C result in poor sinterability, while its inherent brittleness and low fracture toughness severely restrict its further large-scale engineering applications [4]. Moreover, the relatively low electrical conductivity of monolithic B_4_C hinders its processing by electrical discharge machining (EDM), posing challenges for fabricating complex-shaped components [5].

Graphene, a typical two-dimensional carbon nanomaterial, exhibits ultrahigh Young’s modulus, remarkable fracture strength, excellent electrical and thermal conductivity, as well as a large specific surface area [6,7,8,9,10], making it an ideal reinforcing phase for ceramic matrices. Graphene exists in various forms and dimensions, including graphene nanosheets (GNSs), nanoplatelets (GNPs), graphene platelets (GPLs), quantum dots, and nanoribbons [11]. The incorporation of GNPs into B_4_C ceramics not only improves their mechanical properties but also endows the composite with favorable electrical conductivity for EDM processing [12].

Hot pressing (HP), a mature sintering technology with high densification efficiency, has been widely employed to prepare graphene-reinforced ceramic composites. Numerous previous studies have successfully fabricated high-performance ceramic–graphene composites via HP [13,14,15,16,17,18,19,20,21,22]. It has been confirmed that GNPs can effectively toughen B_4_C ceramics through multiple mechanisms, including crack deflection, crack bridging and graphene pull-out [23,24,25,26]. Tan et al. investigated the effect of GNP content on the fracture toughness of B_4_C composites and reported a progressive increase in fracture toughness with increasing GNP volume fraction, reaching a maximum value of 5.26 MPa·m^1/2^ at 4 vol% GNPs [27]. Yin et al. found that the addition of 1.5 wt% graphene enhanced the fracture toughness of B_4_C ceramic by 23.1% and its ballistic resistance by 12.5% compared to monolithic B_4_C ceramic [28]. Meanwhile, relevant studies have also focused on the structural stability of graphene during high-temperature hot pressing, as well as the effects of graphene content and type on the mechanical, electrical, and thermal properties of B_4_C composites [24,29,30].

It is noteworthy that the strengthening and toughening effects of graphene fundamentally rely on its uniform dispersion within the B_4_C matrix. Severe agglomeration of graphene induced by van der Waals forces can introduce numerous structural defects and critical flaws into the composites, thereby deteriorating the mechanical properties. Kovalčíková et al. observed that increasing GPL content led to a decline in Vickers hardness and flexural strength and attributed this degradation to the presence of graphene agglomerates and structural inhomogeneities acting as fracture origins [31]. Alexander et al. reported that excessive and poorly dispersed graphene resulted in the formation of interconnected networks, agglomerates, and interfacial pores, which in turn degraded the properties of B_4_C composites [32].

However, most current studies have paid little attention to the powder homogenization process and dispersion state of graphene, and few investigations have systematically explored the influence of powder preparation technology on the final microstructure and performance of composites. It is well known that GNPs are hydrophobic, rendering their surface chemistry incompatible with many hydrophilic non-oxide ceramic powders. To address this issue, various methods have been reported to modify the surface of graphene or GNPs and to improve their dispersion in composite materials. These approaches mainly involve the functionalization of graphene or graphene oxide surfaces [33] or the use of surfactants, polymers, or aromatic molecules as dispersants [34]. Surfactants not only facilitate the dispersion of graphene oxide in aqueous media but also direct the self-assembly of B_4_C into hybrid architectures [30]. In our previous work, cetyltrimethyl ammonium bromide (CTAB), a cationic surfactant, was adopted to achieve the uniform dispersion of GNPs in B_4_C powder by heterogeneous co-precipitation, demonstrating the feasibility of CTAB for preparing well-dispersed B_4_C–GNP composite powders. Nevertheless, the stabilization mechanisms of CTAB-modified B_4_C–GNP composite powders remain unclear.

This work aims to elucidate the mechanisms of CTAB-assisted heterogeneous co-precipitation for fabricating uniformly dispersed B_4_C–GNP hybrid powders. Subsequently, the effects of GNP content on the microstructure, defect characteristics, and mechanical properties of B_4_C composite ceramics are systematically evaluated, and the corresponding strengthening and toughening mechanisms of B_4_C–GNP composites are discussed.

## 2. Experimental Procedures

### 2.1. Experimental Materials

**Figure 1:**
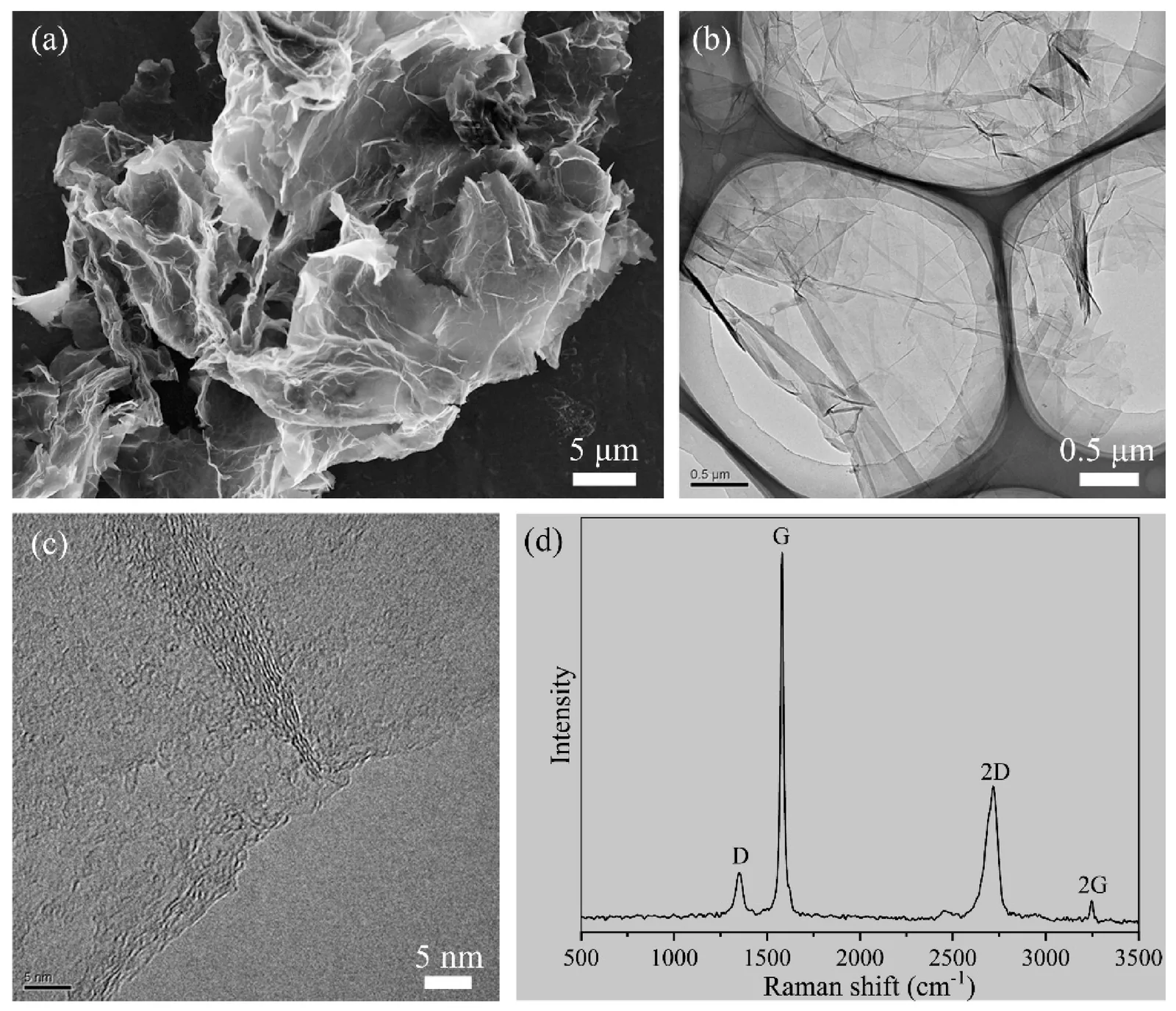
Characteristic microstructure of the as-received GNP powder: (**a**) SEM image, (**b**) TEM image, (**c**) HRTEM image and (**d**) Raman spectrum of GNPs.

B_4_C–GNP composites were prepared using commercially available B_4_C powder (Jingangzuan Boron carbide Co., Ltd., Mudanjiang, China) and GNP powder supplied by Deyang Carbonene Technology Co., Ltd., Deyang, China. The microstructure of the raw GNPs is shown in Figure 1. As can be seen in Figure 1a, the as-received GNPs exist as agglomerates with a size of several tens of micrometers. After ultrasonic treatment in ethanol for 2 h, the morphology of the dispersed GNPs is shown in Figure 1b,c. Under TEM, the GNPs appear as nearly transparent thin sheets, with varying degrees of wrinkles and folds observed on their surfaces, and their thickness is measured to be approximately 4–5 nm. Figure 1d presents the Raman spectrum of the pristine GNP powder. The D band shows a very low intensity relative to the G band, indicating that the raw GNPs contain virtually no significant structural defects. CTAB (cetyltrimethylammonium bromide), used as a dispersant in this study, was purchased from Sigma-Aldrich Corp. (St. Louis, MO, USA).

### 2.2. Preparation of B_4_C–GNP Composite Ceramics

**Figure 2:**
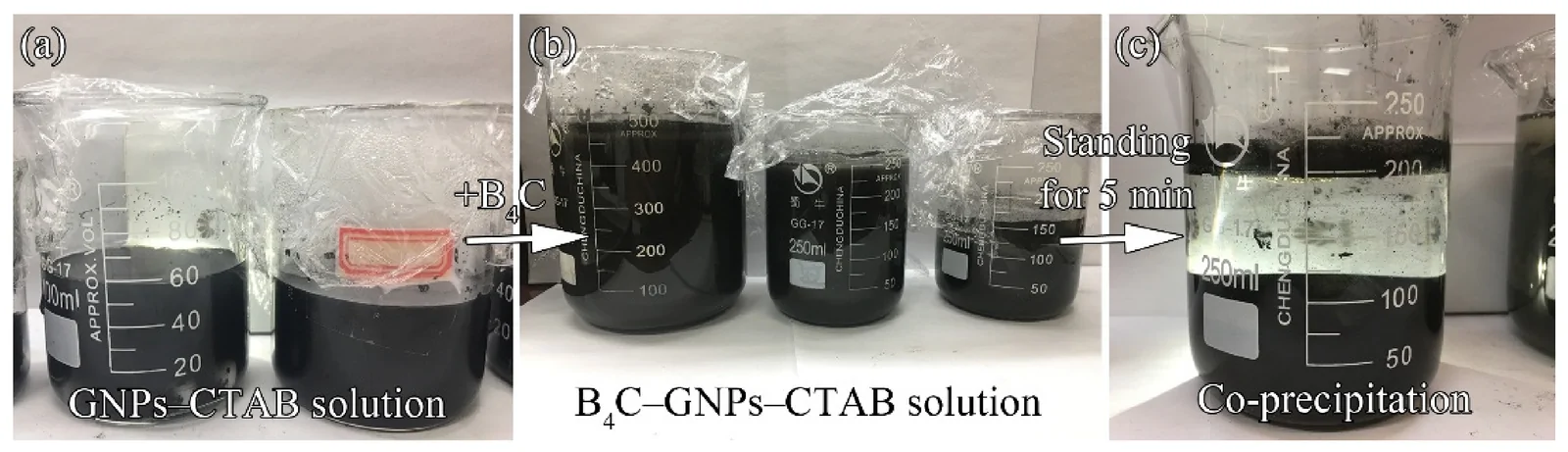
Photographs of (**a**) the homogeneously dispersed GNP–CTAB aqueous solution, (**b**) the uniformly dispersed B_4_C–GNP–CTAB slurry, and (**c**) the stratified solution.

First, different amounts of GNPs (0 wt%, 0.5 wt%, 1 wt%, 2 wt%, and 4 wt%) were directly added to a pre-prepared 1 wt% CTAB aqueous solution (pH = 6.5) and magnetically stirred for 2 h to form a homogeneously dispersed GNP–CTAB solution (Zeta potential: +41.8 mV), as shown in Figure 2a. Subsequently, B_4_C powder was gradually introduced into the GNP–CTAB solution, and the mixture was stirred for an additional 2 h to form a uniformly dispersed B_4_C–GNP–CTAB slurry, as shown in Figure 2b. Upon settling for 2–5 min, the mixed solution underwent stratification, resulting in the formation of a transparent supernatant and a precipitated bottom layer, as shown in Figure 2c. The resulting precipitate was then freeze-dried to produce a uniform B_4_C–GNP powder mixture.

Finally, 16 g of the as-prepared B_4_C–GNP powder mixture was loaded into a ϕ 48 mm graphite die and sintered in a hot-pressing furnace (916G-G Press, Thermal Technology Inc., Santa Rosa, CA, USA) at 1950 °C for 1 h under a uniaxial pressure of 30 MPa in an argon atmosphere. The heating rate was set at 20 °C/min from room temperature (25 °C) to 1200 °C under vacuum, followed by 10 °C/min to 1950 °C under argon. An initial pressure of 6 MPa was applied and gradually increased to 30 MPa as the temperature rose from 1200 °C to 1950 °C, and this pressure level was maintained until the sintering was completed, after which the furnace was cooled to room temperature.

### 2.3. Experimental Methods

For microstructural characterization and mechanical property evaluation, specimens were sectioned using electrical discharge machining (EDM), ground with a diamond wheel, and finally polished with a 0.25 μm diamond suspension to minimize residual surface stresses. The density of each sintered sample was determined via the Archimedes method using deionized water. Theoretical densities were calculated based on the rule of mixtures, adopting values of 2.52 g/cm^3^ for B_4_C and 2.2 g/cm^3^ for GNPs. Phase identification was performed by X-ray diffraction (XRD, Rigaku Ultima III, Tokyo, Japan). The microstructural features and GNP distribution within the B_4_C matrix were examined using scanning electron microscopy (SEM, Hitachi 3400, Naka, Japan). The morphology and thickness of the as-received GNPs were characterized by both SEM and transmission electron microscopy (TEM, JEM-2100 F, Akishima, Tokyo, Japan), operated at an accelerating voltage of 200 kV.

Vickers hardness (HV) was evaluated using an indentation tester (Wolpert 430SVD, Wilson Instruments, Norwood, MA, USA) under a load of 9.8 N applied for 15 s, and the hardness value was taken as the average of ten points. Three-point bending tests were conducted on an electro-mechanical universal testing machine (MTS-810, MTS Systems Corporation, Eden Prairie, MN, USA) with a crosshead speed of 0.5 mm/min, a support span of 30 mm, and specimen dimensions of 3 mm × 4 mm × 36 mm. The room-temperature fracture toughness (KIC) was assessed using the single-edge notched beam (SENB) method with an outer span of 20 mm and a displacement rate of 0.05 mm/min. Specimens for toughness testing were machined to dimensions of 2.5 mm × 5 mm × 25 mm, featuring a notch of 0.2 mm in width and 2.5 mm in depth. To prevent edge-initiated failures, both edges of the tensile surface were cut at a 45° angle, and at least five runs were conducted for each test.

## 3. Results and Discussion

### 3.1. The Dispersion Mechanisms of GNPs in B_4_C Powder

**Figure 3:**
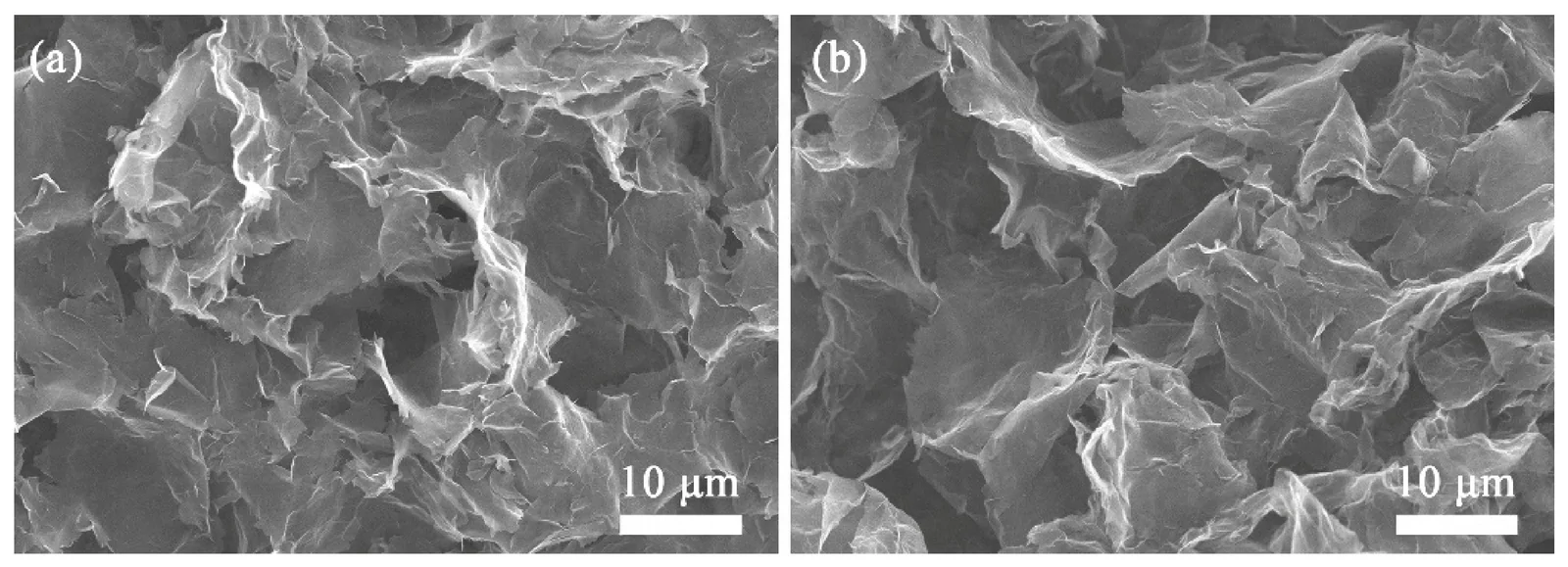
Morphology images of GNPs modified with CTAB. (**a**) Supernatant and (**b**) precipitate after centrifugation.

It is well established that GNPs consist of multilayer graphene, with in-plane carbon atoms covalently bonded and rich in π electrons [35]. This intrinsic electronic structure endows GNPs with hydrophobic characteristics. As a result, when subjected to ultrasonication in deionized water, GNPs inevitably float on the water surface, failing to achieve stable dispersion. When CTAB is added to water, the surface tension of the water decreases, and the wettability and dispersion capability of GNPs are remarkably improved. Figure 3 shows the morphology of CTAB-modified GNPs. Unlike pristine GNPs, which appear as spherical agglomerates, both the supernatant (in Figure 3a) and the centrifugal precipitate (in Figure 3b) of the modified GNPs exhibit a lamellar structure.

**Figure 4:**
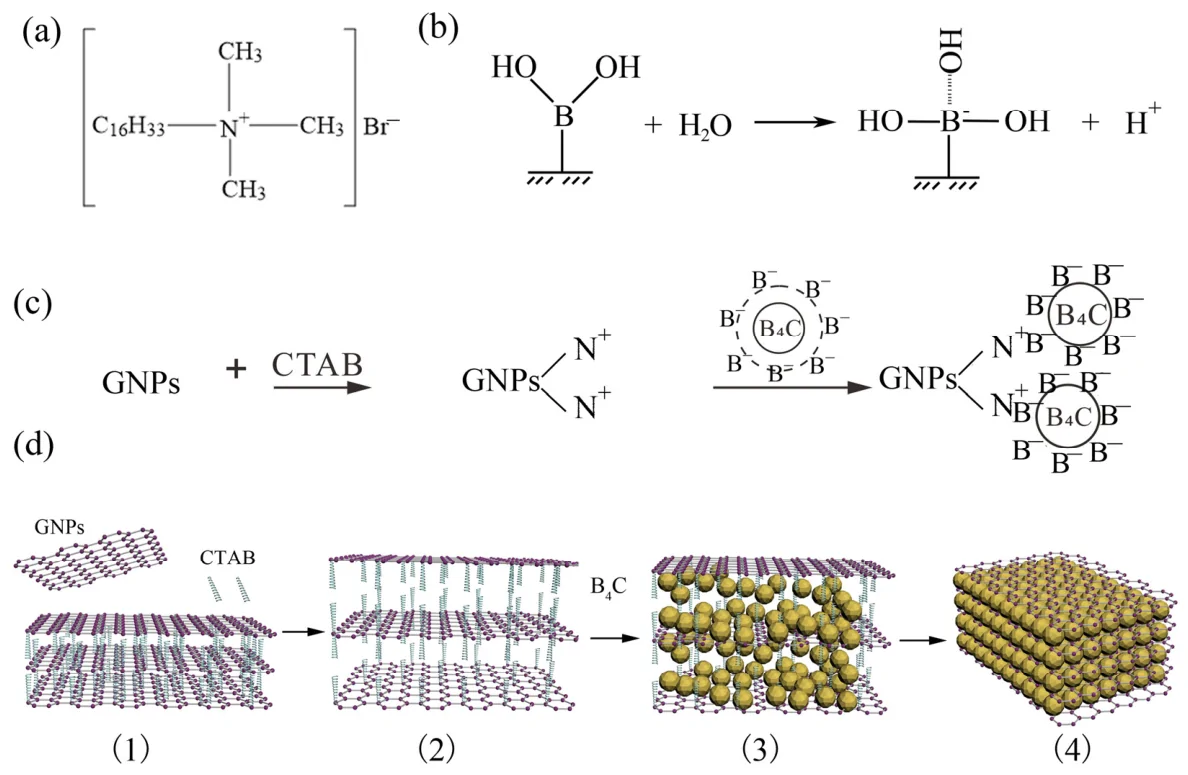
Schematic illustration of the heterogeneous co-precipitation process for fabricating uniformly dispersed B_4_C–GNP hybrids using a CTAB surfactant. (**a**) Chemical structure of CTAB, (**b**) schematic depiction of the B_4_C particle surface in an aqueous environment, (**c**) proposed formation mechanism of B_4_C–GNP hybrids, and (**d**) the detailed assembly process. The process involves four stages. First, CTAB modifies the GNP surface, rendering it positively charged. Second, the CTAB layer undergoes a series of structural transitions from an ordered bilayer to a more fluid bilayer and finally to an interdigitated monolayer. Third, negatively charged B_4_C particles and positively charged GNPs undergo heterogeneous co-precipitation through electrostatic interactions. Fourth, alternating layers of B_4_C and GNP are assembled into layered B_4_C–GNP hybrid structures.

Figure 4 illustrates the formation mechanism of B_4_C–GNP hybrids using CTAB as a surfactant. The chemical structure of CTAB is shown in Figure 4a, while the surface state of B_4_C particles in water is schematically represented in Figure 4b. As illustrated in Figure 4c, the formation mechanism of the B_4_C–GNP hybrids can be rationalized as a two-step process. Initially, the GNPs were functionalized with CTAB, a cationic surfactant, which imparted a positive surface charge to the GNPs. Subsequently, the positively charged GNPs were heterogeneously co-precipitated with the negatively charged B_4_C particles via electrostatic interactions, thereby forming stable B_4_C–GNP hybrid composites.

The detailed assembly process consists of four sequential steps. First, when GNPs are gradually introduced into the CTAB solution, hydrophobic interactions between the alkyl tail of CTAB and the GNP surface, together with cation–π electron interactions between the quaternary ammonium head group and GNPs, drive the adsorption of CTAB molecules onto the GNP surface with the alkyl tail oriented inward and the head group outward. This CTAB assembly renders the GNPs positively charged [36]. The increased steric hindrance between GNPs counteracts the van der Waals forces and prevents re-aggregation. Concurrently, ultrasonication of GNPs in the presence of CTAB promotes their exfoliation into thin graphene flakes. Second, driven by electrostatic repulsion among the quaternary ammonium head groups, the CTAB layer undergoes a structural transition from an ordered bilayer to a more fluid bilayer and eventually to an interdigitated monolayer, which is consistent with the model proposed by Meng et al. [37]. Third, the surface of B_4_C powder typically contains impurities such as a boron oxide coating. In aqueous solution, negatively charged [B(OH)_3_]^−^ groups form and adsorb onto the B_4_C particle surfaces [38,39]. When B_4_C particles are introduced into the GNP–CTAB solution, the negatively charged B_4_C assembles with the positively charged GNPs through electrostatic interactions. Finally, alternating layers of B_4_C and GNP are assembled into layered structures after the removal of the surfactant [40].

### 3.2. The Influence of GNP Content on the Microstructure of B_4_C Composites

**Figure 5:**
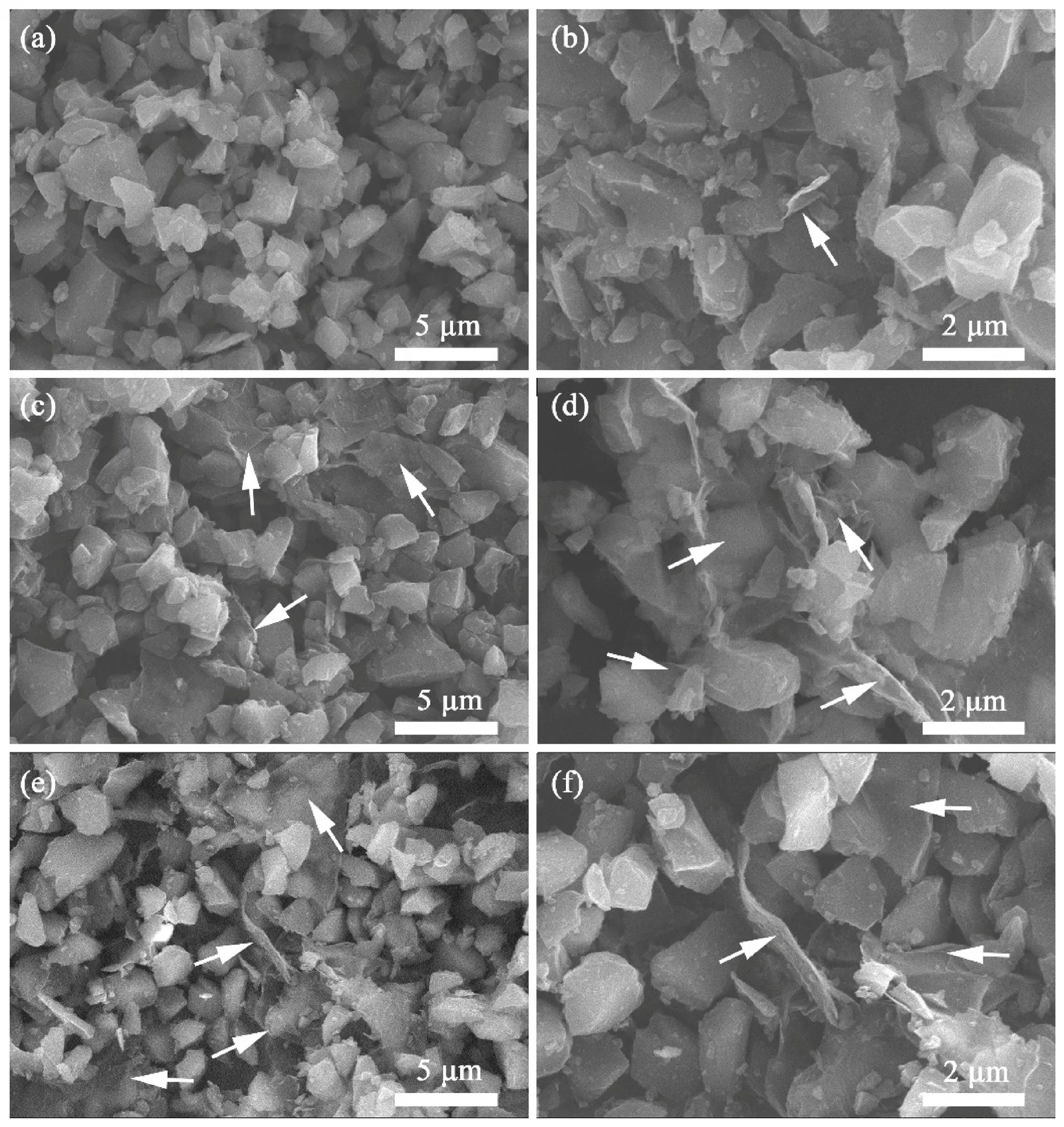
SEM images of B_4_C–GNP powder mixtures. (**a**) 0.5 wt% GNPs, (**b**) 1 wt% GNPs, (**c**) 2 wt% GNPs, (**d**) higher magnification of (**c**), (**e**) 4 wt% GNPs, (**f**) higher magnification SEM of (**e**).

SEM images of the prepared B_4_C powder mixtures are presented in Figure 5. When the GNP content is 0.5 wt%, a few GNPs can be observed within the B_4_C matrix. As the GNP content increases to 1 wt%, a small number of GNPs become visible. When the GNP content is further increased to 2 wt% or above, the GNPs are uniformly distributed throughout the B_4_C matrix, as indicated by the white arrows in Figure 5c–f. Notably, the GNPs are well separated from one another, and their edges exhibit a corrugated morphology within the B_4_C matrix, as shown in Figure 5d.

**Figure 6:**
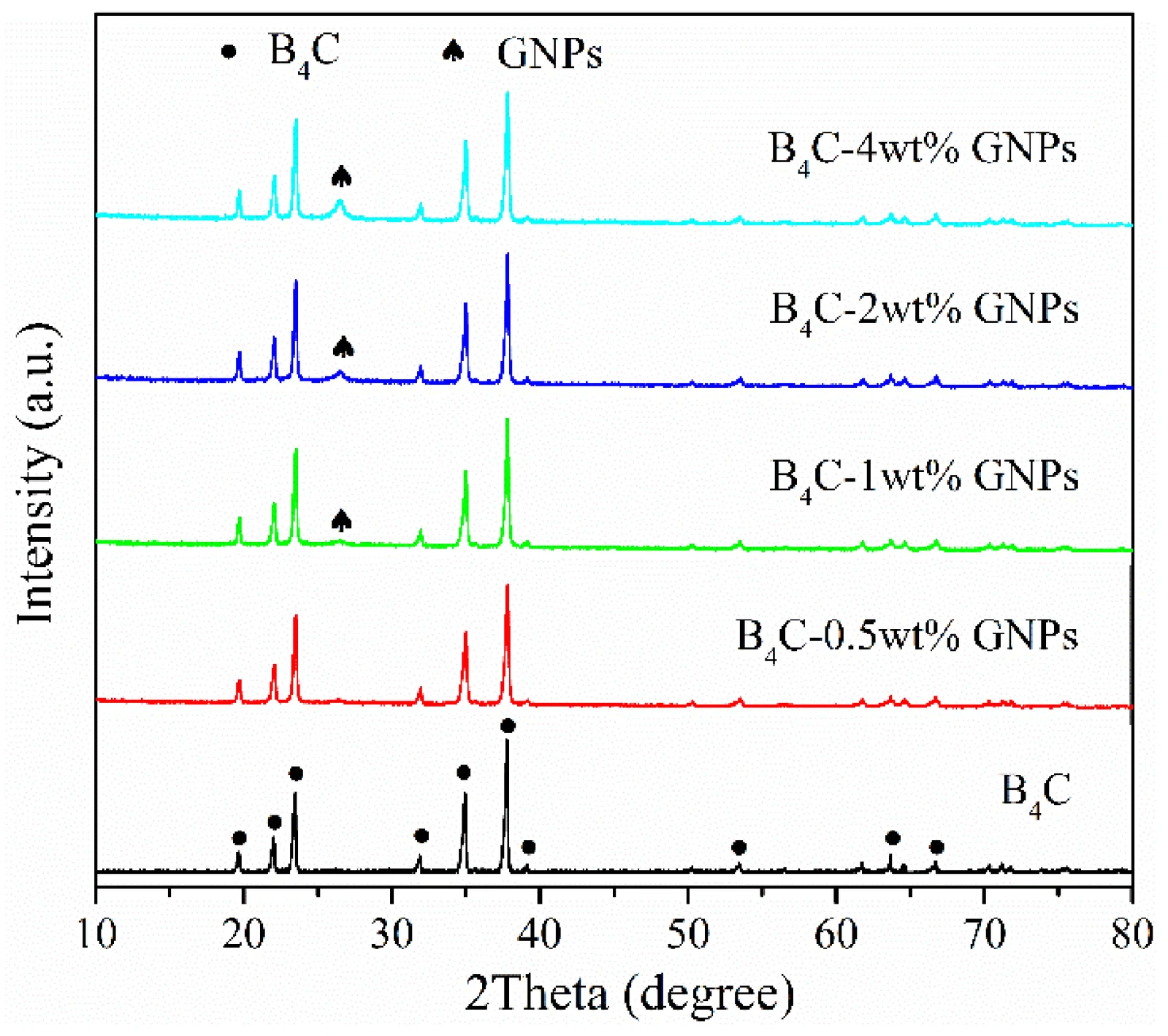
XRD patterns of B_4_C–GNP powder mixtures.

Figure 6 presents the XRD patterns of the B_4_C–GNP powder mixtures, confirming the presence of only B_4_C and GNPs, with no evidence of secondary phases. The characteristic GNP diffraction peak, typically observed at approximately 26°, is not detected at a GNP content of 0.5 wt%, likely because it falls below the detection limit of XRD. When the GNP content increases to 1 wt%, a weak GNP peak becomes detectable, and its intensity rises progressively with further increases in GNP loading.

**Figure 7:**
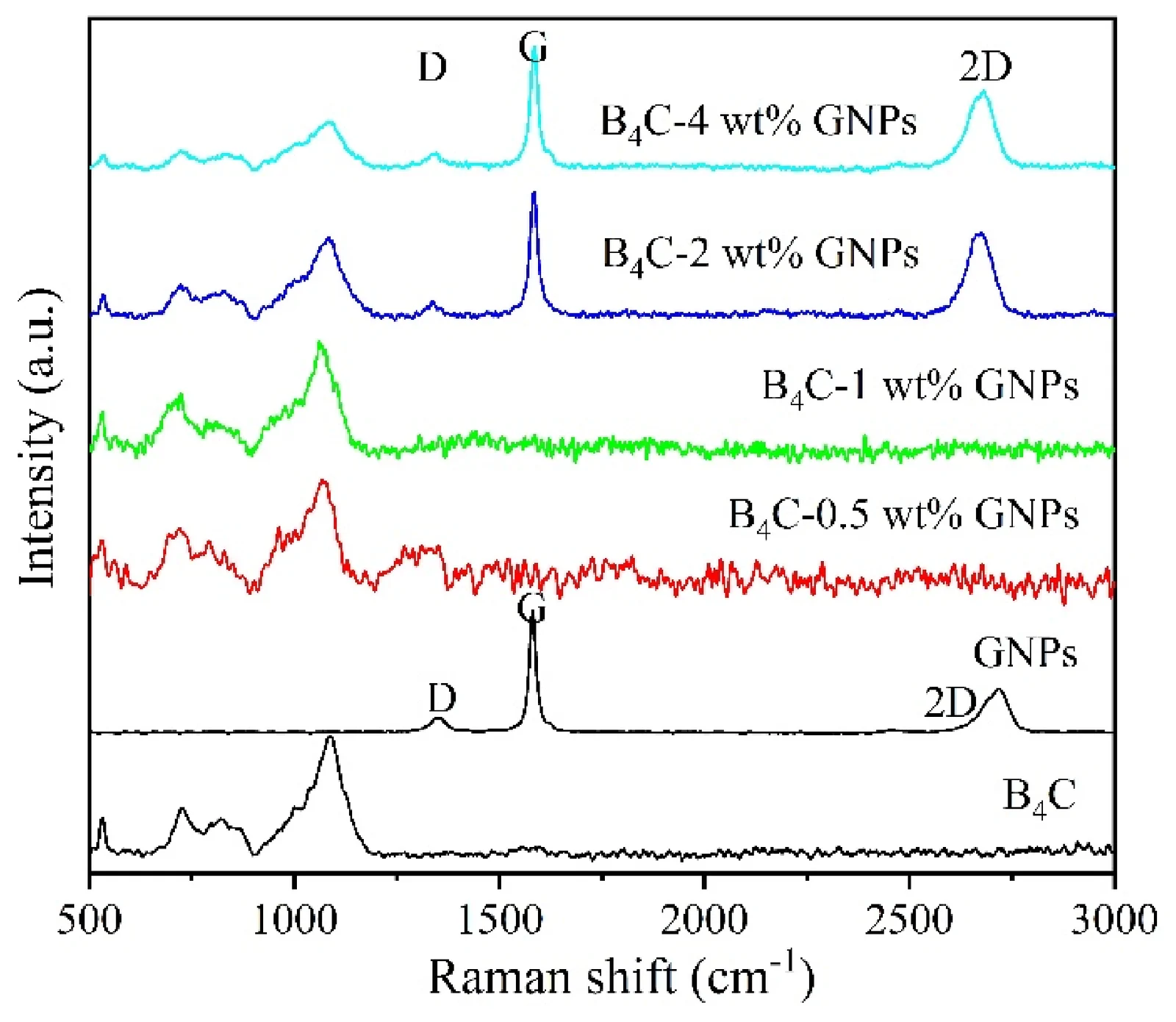
Raman spectra of B_4_C–GNP powder mixtures.

Figure 7 presents the Raman spectra of the B_4_C–GNP powder mixtures. The D band at 1350 cm^−1^ signifies the presence of lattice disorder, and its intensity is proportional to the level of defects in the samples. The G band at approximately 1580 cm^−1^ corresponds to the crystallization peak of graphene, which is strongly associated with the in-plane C–C symmetric stretching vibrations. The degree of structural defects in graphene is often characterized by the intensity ratio of the D and G bands (I_D_/I_G_), which increases with increasing defect density. Meanwhile, the intensity of the 2D band reflects the number of graphene stacking layers, and the I_2D_/I_G_ ratio is commonly used to determine the number of graphene sheets; this ratio is approximately 4 for single-layer graphene and decreases with increasing layer number [41,42]. As shown in Figure 7, when the GNP content is below 2 wt%, no characteristic GNP peaks are observed. However, when the GNP content exceeds 2 wt%, a weakened D band and a distinct 2D band become detectable, confirming that the B_4_C–GNP powder mixtures prepared by the heterogeneous co-precipitation method retain the layered structure of GNPs.

**Figure 8:**
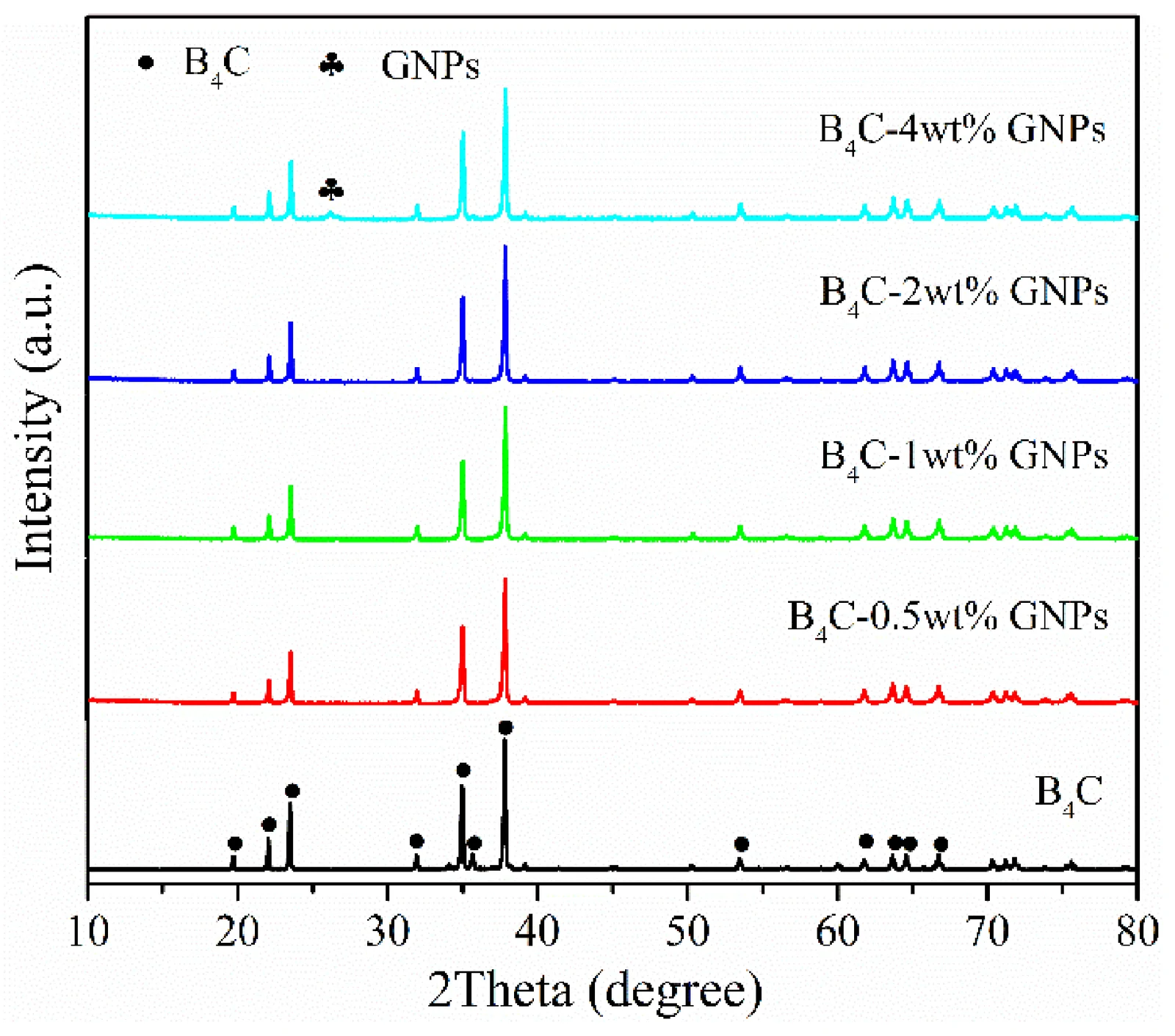
XRD patterns of B_4_C–GNP composites.

Figure 8 presents the XRD patterns of the B_4_C–GNP composites. No diffraction peaks attributable to GNPs are observed when the GNP content is below 2 wt%. In contrast, the characteristic GNP reflections are clearly discernible in the corresponding powder mixtures containing 0.5 and 1 wt% GNPs. This discrepancy indicates that the effective GNP content in the sintered B_4_C composites is reduced relative to the initial loading. The reduction is plausibly attributed to the reaction between GNPs and B_2_O_3_, which is well-documented as a surface residue on B_4_C particles originating from the carbothermal reduction synthesis route. This reaction has been extensively reported in the literature [32].

**Figure 9:**
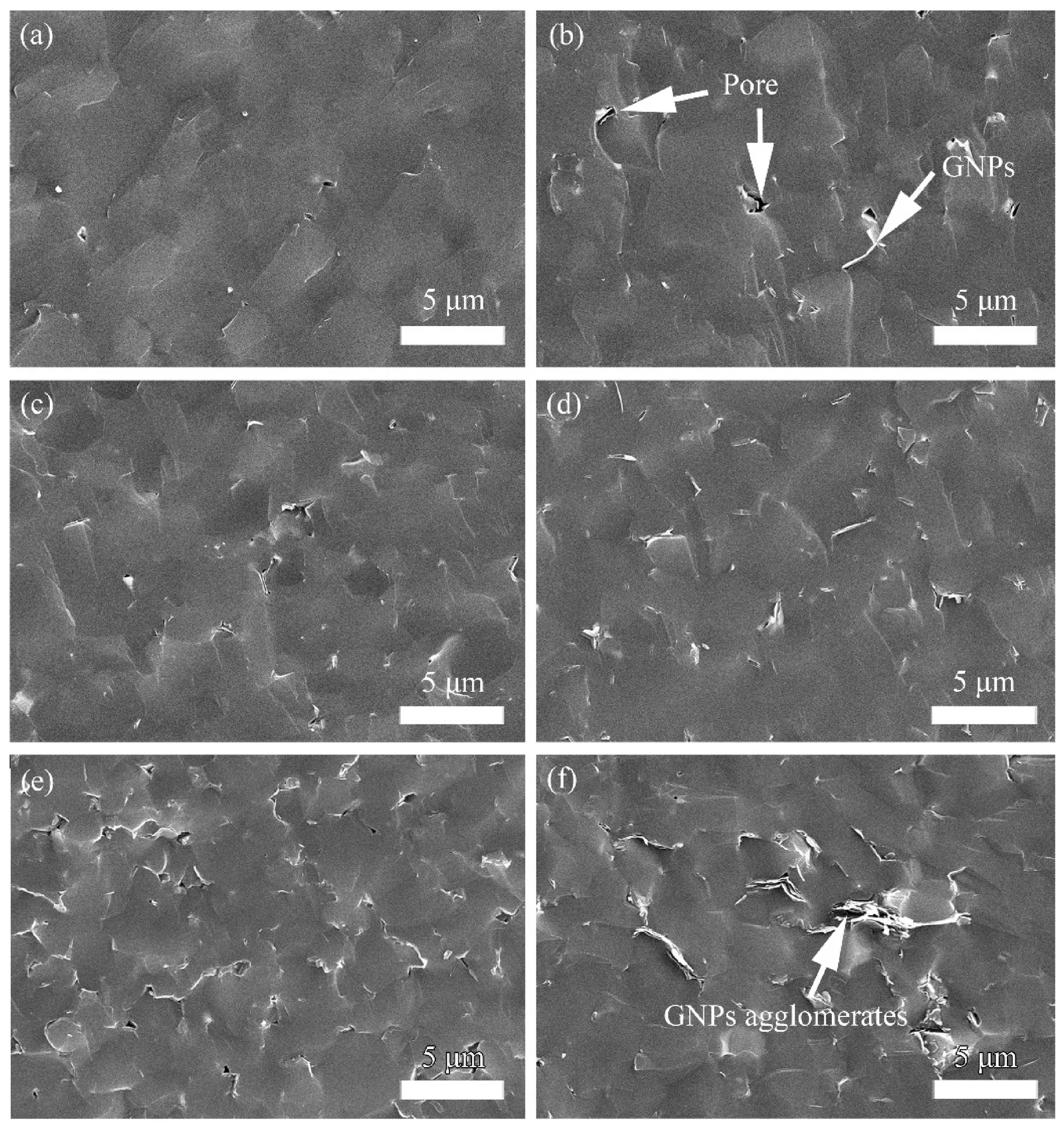
SEM images at (**a**) 0wt%, (**b**) 0.5 wt% GNPs, (**c**) 1 wt% GNPs, (**d**) 2 wt% GNPs, (**e**) 4 wt% GNPs and (**f**) GNP agglomerates in the B_4_C–4 wt% GNP composite.

Figure 9 shows SEM images of the fracture surfaces of B_4_C–GNP composites with varying GNP content. The fracture surface of monolithic B_4_C is relatively smooth with few residual pores, as shown in Figure 9a, suggesting a typical transgranular fracture mode. At a GNP content of 0.5 wt%, only a limited number of GNPs are discernible within the B_4_C matrix, as shown in Figure 9b. This is attributed not only to the low GNP loading but also to the thin nature of the GNPs, which are oriented perpendicular to the fracture surface and thus prone to rupture under stress [43].

When the GNP content is increased to 1 wt% and 2 wt%, the composites exhibit dense microstructures free of observable porosity, with GNPs uniformly dispersed throughout the B_4_C matrix, as shown in Figure 9c,d. Pulled-out GNPs are observed around B_4_C grain boundaries, randomly protruding from the fracture surface, with no preferential orientation relative to the hot-pressing axial direction. This observation contrasts with previous reports [23] and is possibly related to the relatively small size of the GNPs after sintering, which will be further investigated in future work. At a GNP content of 4 wt%, the fracture surfaces generally display homogeneous microstructures, indicating uniform GNP distribution, as shown in Figure 9e. The GNPs consist of nanoplatelets approximately 30 nm in thickness and 2–4 μm in length. However, locally, tiny agglomerates with a thickness of about 50–100 nm are observed, as shown in Figure 9f, formed by overlapping GNPs, as indicated by the white arrow.

**Figure 10:**
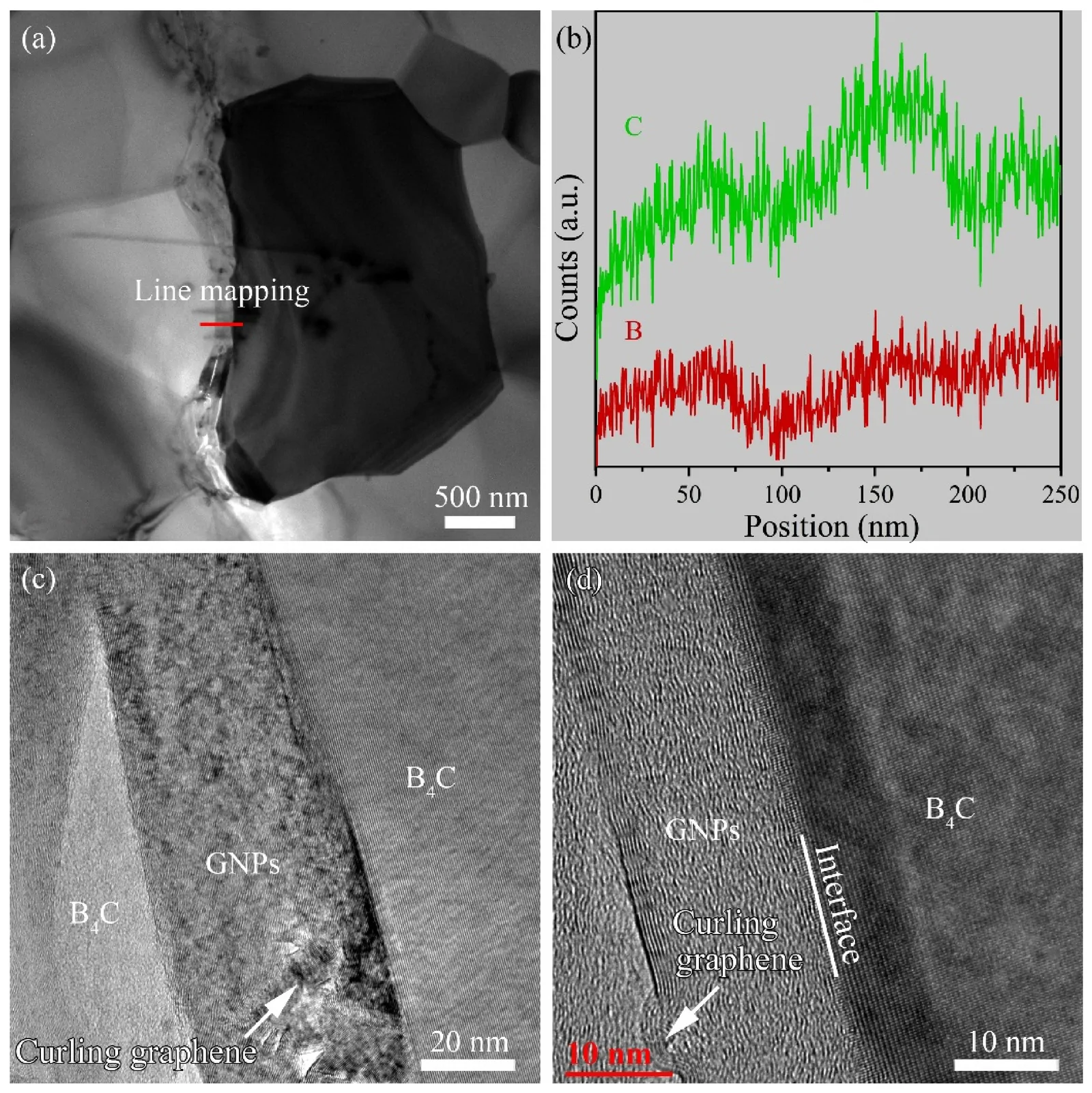
(**a**) TEM image of the B_4_C–4 wt% GNP composite, (**b**) EDS analysis of the GNPs in the B_4_C matrix, (**c**) enlarged TEM image from (**a**), and (**d**) HRTEM image of B_4_C lattice fringes.

Figure 10 shows TEM and HRTEM images of the B_4_C–4 wt% GNP composite. As shown in Figure 10a, curved GNPs are located at the grain boundaries of B_4_C and tightly wrap around the B_4_C grains. The presence of GNPs is confirmed by EDS line scan analysis, as displayed in Figure 10b. Figure 10c provides a higher magnification TEM image of Figure 10a, revealing that the GNP layers are primarily composed of multilayered graphene sheets arranged in parallel, with some curling observed at certain positions, as indicated by the white arrows. Furthermore, the HRTEM image in Figure 10d reveals a clean and well-bonded interface between the GNPs and the B_4_C matrix that is free of any detectable secondary phases or impurities. Such a pristine interfacial structure provides a fundamental basis for the enhanced mechanical properties of the composite.

### 3.3. The Influence of GNP Content on Mechanical Properties of B_4_C Composites

Table 1 summarizes the mechanical properties of B_4_C composites with varying GNP content. The monolithic B_4_C exhibits a relative density of 98.67%, a Vickers hardness of 32.6 GPa, a flexural strength of 388 MPa, and a fracture toughness of 2.99 MPa·m^1/2^. With the incorporation of GNPs, the relative density initially increases, reaching a maximum of 99.65% at 1 wt% GNPs, and then gradually decreases to 98.12% at 4 wt% GNPs. The initial densification enhancement is attributed to the self-lubricating effect of carbon, which facilitates particle rearrangement, and to the removal of surface boron oxide via interfacial reactions during sintering [22]. However, excess GNPs form interconnected networks that hinder B_4_C particle diffusion and create interfacial porosity owing to differential shrinkage between GNPs and the B_4_C matrix.

Similarly, the Vickers hardness and flexural strength exhibit comparable trends, increasing from 32.6 GPa and 388 MPa for monolithic B_4_C to peak values of 33.5 GPa and 488 MPa at 1 wt% GNPs, followed by a decline to 31.2 GPa and 439 MPa at 4 wt% GNPs, respectively. In contrast, the fracture toughness of B_4_C ceramics is significantly enhanced by GNP addition. It increases from 2.99 MPa·m^1/2^ for monolithic B_4_C to a maximum of 4.89 MPa·m^1/2^ at 2 wt% GNPs, representing a remarkable improvement of approximately 63.5%. Even at a higher GNP content of 4 wt%, the fracture toughness remains at a high level of 4.52 MPa·m^1/2^.

The initial increase and subsequent decrease in hardness of B_4_C composites are closely related to the variation in relative density. Moreover, the hardness evolution is also influenced by grain boundary sliding and the deformation behavior of GNPs. When the applied load is transferred from the B_4_C matrix to the GNPs, it is resolved into in-plane and out-of-plane stress components. The in-plane stress induces sliding or even cleavage of the GNPs, while the out-of-plane stress leads to their deformation. Both effects facilitate localized deformation and consequently result in a reduction in hardness [19].

Furthermore, the improvement in flexural strength can be ascribed to three synergistic effects, including the higher relative density, the uniform distribution of GNPs, and the resulting high interfacial density. The abundant interfaces impede dislocation movement [25], which plays a critical role in strengthening the B_4_C composites. However, further increasing the GNP content leads to a decline in flexural strength, primarily due to the GNP agglomeration shown in Figure 9f. These agglomerates act as large critical flaws and serve as fracture origins, thereby reducing the fracture strength [13,44,45].

### 3.4. Toughening Mechanisms of GNPs in B_4_C Composites

**Figure 11:**
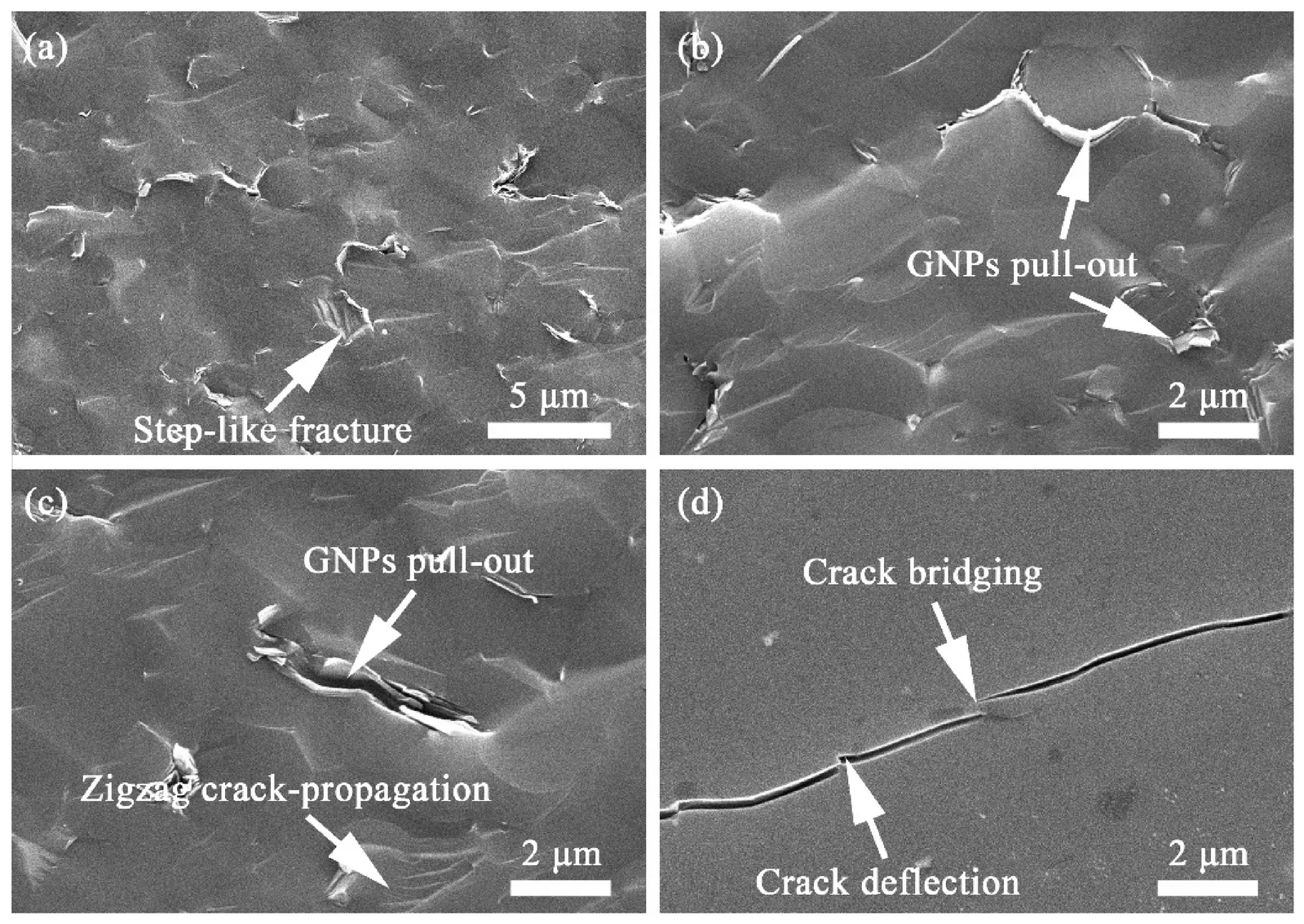
Toughening mechanisms in B_4_C–GNP composites. (**a**) Step-like fracture, (**b**) GNP pull-out, (**c**) the pores left after the pull-out of GNPs and zigzag crack propagation, and (**d**) crack bridging and deflection.

In the fracture surface and Vickers indentation cracks of B_4_C–GNP composites, multiple toughening mechanisms are observed, including crack deflection, crack bridging, GNP pull-out, step-like fracture, and zigzag crack propagation. The step-like fracture shown in Figure 11a represents an intragranular crack deflection pattern. This stepped crack propagation path effectively dissipates substantial fracture energy, thereby enhancing the fracture toughness of the material. The pull-out of GNPs is evident in Figure 11b, and the resulting pits left after pull-out are shown in Figure 11c. During densification, interfacial stress promotes the bending and intergranular embedding of GNPs, resulting in close contact with B_4_C grains. This close contact enhances the anchorage of GNPs and improves interfacial adhesion. As shown in Figure 11b, GNPs wrapped around grain boundaries provide extensive interfacial areas, generating elevated interfacial friction against pull-out from the B_4_C matrix. Consequently, the energy required for GNP pull-out exceeds that for other nanofibers [14,46]. The pull-out process continues until the interfacial shear strength limit is reached, and the toughening effect is largely governed by the interfacial friction between GNPs and the matrix. The enhancement of interfacial friction therefore contributes to improved fracture toughness.

The multilayered structure of GNPs and the pores left after their pull-out are presented in Figure 11c. The outermost layers adhere firmly to the ceramic matrix during densification and sustain the applied stress owing to the elasticity of GNPs. During pull-out, the stress transfers from the outermost to the inner layers and can propagate over long distances through interlayer slippage before detachment. This delayed detachment induces a slip–stick effect and generates complex interlayer frictional forces, as addressed in previous theoretical studies [47,48]. Given the high specific surface area of GNPs, the slip–stick effect extends across a broad region, and additional sliding interfaces are formed during interlayer movement. Overcoming the interlayer friction and enabling continued sliding between adjacent layers before final detachment consumes substantial energy, thereby enhancing fracture toughness. Furthermore, crack deflection and crack bridging, as illustrated in Figure 11d, together with the aforementioned toughening mechanisms, contribute significantly to the improvement in fracture toughness.

## 4. Conclusions

B_4_C–GNP composites were successfully fabricated by hot pressing using heterogeneously co-precipitated powder mixtures with CTAB as the surfactant. CTAB-induced electrostatic assembly effectively resolves GNP agglomeration and constructs uniform, layered hybrid microstructures. The optimal GNP content was 1 wt%, at which the composite exhibited a relative density of 99.65%, a Vickers hardness of 33.5 GPa, and a flexural strength of 488 MPa. In contrast, the fracture toughness peaked at 4.89 MPa·m^1/2^ with 2 wt% GNPs, marking a 63.5% increase over monolithic B_4_C. Moderate GNP incorporation improves the densification and overall mechanical properties of B_4_C composites through multiple toughening mechanisms, including crack deflection, crack bridging, GNP pull-out, step-like fracture, and zigzag crack propagation. This powder modification strategy enables uniform GNP dispersion and facilitates the fabrication of B_4_C composites with balanced and optimized mechanical properties.

## Figures and Tables

**Table 1:** Mechanical properties of B_4_C–GNP composites.

Samples	Relative Density (%)	Vickers Hardness (GPa)	Flexural Strength (MPa)	Fracture Toughness (MPa·m^1/2^)
B_4_C	98.67	32.6 ± 0.5	388 ± 15	2.99 ± 0.2
B_4_C–0.5 wt% GNPs	99.21	32.8 ± 0.4	452 ± 25	3.75 ± 0.3
B_4_C–1 wt% GNPs	99.65	33.5 ± 0.5	488 ± 18	4.58 ± 0.2
B_4_C–2 wt% GNPs	99.37	33.1 ± 0.6	461 ± 32	4.89 ± 0.3
B_4_C–4 wt% GNPs	98.12	31.2 ± 0.6	439 ± 40	4.52 ± 0.4

## Data Availability

The original contributions presented in this study are included in the article. Further inquiries can be directed to the corresponding authors.
